# Integrative analysis of indirect calorimetry and metabolomics profiling reveals alterations in energy metabolism between fed and fasted pigs

**DOI:** 10.1186/s40104-018-0257-x

**Published:** 2018-05-16

**Authors:** Hu Liu, Yifan Chen, Dongxu Ming, Ji Wang, Zhen Li, Xi Ma, Junjun Wang, Jaap van Milgen, Fenglai Wang

**Affiliations:** 1 State Key Laboratory of Animal Nutrition, China Agricultural University, Beijing, 100193 China; 2 State Key Laboratory of Plant Physiology and Biochemistry, College of Biological Sciences, China Agricultural University, Beijing, 100193 China; 3 INRA, UMR Pegase, 35590 Saint-Gilles, France

**Keywords:** Fasting, Growing pig, Indirect calorimetry, Metabolomics, Plasma

## Abstract

**Background:**

Fasting is a simple metabolic strategy that is used to estimate the maintenance energy requirement where the energy supply for basic physiological functions is provided by the mobilization of body reserves. However, the underlying metabolic components of maintenance energy expenditure are not clear. This study investigated the differences in heat production (HP), respiratory quotient (RQ) and plasma metabolites in pigs in the fed and fasted state, using the techniques of indirect calorimetry and metabolomics.

**Methods:**

Nine barrows (45.2 ± 1.7 kg BW) were fed corn-soybean based meal diets and were kept in metabolism crates for a period of 14 d. After 7 d adaptation, pigs were transferred to respiratory chambers to determine HP and RQ based on indirect calorimetry. Pigs were fed the diet at 2,400 kJ ME/(kg BW^0.6^·d) during d 8 to 12. The last 2 d were divided into 24 h fasting and 48 h fasting treatment, respectively. Plasma samples of each pig were collected from the anterior vena cava during the last 3 d (1 d while pigs were fed and 2 d during which they were fasted). The metabolites of plasma were determined by high-resolution mass spectrometry using a metabolomics approach.

**Results:**

Indirect calorimetry analysis revealed that HP and RQ were no significant difference between 24 h fasting and 48 h fasting, which were lower than those of fed state (*P* <  0.01). The nitrogen concentration of urine tended to decrease with fasting (*P* = 0.054). Metabolomics analysis between the fed and fasted state revealed differences in 15 compounds, most of which were not significantly different between 24 h fasting and 48 h fasting. Identified compounds were enriched in metabolic pathways related to linoleic acid metabolism, amino acid metabolism, sphingolipid metabolism, and pantothenate and CoA biosynthesis.

**Conclusion:**

These results suggest that the decreases in HP and RQ of growing pigs under fasting conditions were associated with the alterations of linoleic acid metabolism and amino acid metabolism. The integrative analysis also revealed that growing pigs under a 24-h fasting were more appropriate than a 48-h fasting to investigate the metabolic components of maintenance energy expenditure.

## Background

Maintenance corresponds to the basal energy requirements for supporting body function, body temperature, and normal physical activity at a time when there is no net gain or loss of energy [1]. Though it is vital to the organism, little is known of the metabolic components of maintenance energy expenditure [2]. For nearly five decades, the fasting method has been used to estimate maintenance. During fasting, pigs mobilize body reserves resulting in a negative energy balance and, during a short duration of fasting, the fasting heat production may be related to the maintenance energy expenditure in the fed state [3, 4]. Therefore, the fasting metabolism may, to some extent reflect, the metabolic components of maintenance in pigs [5]. A comparison between the fed and the fasted state will contribute to identify specific metabolic indicator of maintenance in pigs [6]. However, the duration of fasting has not been consistent in different studies [7, 8]. A series of studies have demonstrated that fasting duration plays a role in blood biochemistry and transcriptional regulation of metabolic genes. During the short-term fasting, plasma non-esterified fatty acids (NEFA) increased, while leptin and insulin concentrations were reduced [9, 10]. In addition, pyruvate dehydrogenase in rat muscle and fatty acid synthase in rat adipose tissue decreased after 1 d of fasting [11]. During the long-term fasting, plasma glucose level was reduced by 48 h of fasting in fasted pigs and dairy cattle [10]. It was noted that the transcriptions of genes involved in the fatty acid oxidation began to decrease in 3-d fasted rat liver [11]. Thus, it is important to investigate the effect of fasting duration on plasma metabolic profiling to understand deeply the fasting metabolism.

Indirect calorimetry is a noninvasive method to study energy expenditure by respiratory gas exchange analysis [12]. However, changes in heat production (HP) and in the respiratory quotient (RQ) after fasting based on indirect calorimetry are a phenotypic reaction of an organism’s metabolism, as it adapts to a different physiological and nutritional condition [13]. Fasting metabolism is associated with many metabolic changes that occur in the body when the animal has to rely on its body reserves to sustain maintenance [14, 15]. Typically, the NEFA level in plasma increases 12 h after fasting in growing pigs [9]. Also, important increases in the ratio between NEFA and glycerol can be observed after prolonged fasting from 12 h to 36 h [6].

Plasma can be used as an accessible metabolic footprint that provides a picture of the metabolic events in the organisms and may reveal changes in metabolic pathways under various physiological or nutritional conditions [16, 17]. The metabolome is defined as the collection and global analysis of all small molecular metabolites generated in a cell, organ or organism [18]. Rubio-Aliaga et al. [6] used metabolomics of prolonged fasting in humans to reveal new catabolic markers. Thus, metabolomics is an ideal tool to explore the effect of fasting on plasma metabolites that result from synthetic and catabolic processes in growing pigs [19, 20].

However, what the association do the plasma metabolites of pigs have with alterations of components of energy expenditure or substrate oxidation pattern under fasting duration, to our knowledge, is not known clearly. We hypothesized that the changes in HP and RQ of fasted pigs were modulated by potential metabolic pathways related to energy metabolism. Therefore, the objective of present study was to investigate the effect of fasting treatment in growing pigs on the alterations in energy metabolism based on the integrative analysis of indirect calorimetry and metabolomics profiling.

## Methods

### Animal, diets and experimental procedures

The experimental protocol used in the present study was approved by the Institutional Animal Care and Use Committee at China Agricultural University. Nine growing barrows (Duroc × Landrace × Yorkshire), with an average initial BW of 45.2 ± 1.7 kg, were selected from the Fengning Swine Research Unit of China Agricultural University (Hebei, China). The experiment was conducted at the Laboratory of Animal Metabolism of China Agricultural University (Beijing, China). The basal diet (Table 1) was formulated based on corn and soybean meal.

**Table 1:** Composition and nutrient analysis of the basal diet, as-fed basis

Items	Basal diet
Ingredients, %	
Corn	74.95
Soybean meal	22.23
Dicalcium phosphate	0.70
Limestone	0.70
Salt	0.35
Vitamin and mineral premix^a^	0.50
Lysine HCl	0.39
Methionine	0.05
Threonine	0.11
Tryptophan	0.02
Calculated chemical composition,%^b^	
SID Lysine	0.98
SID Methionine	0.28
SID Tryptophan	0.17
SID Threonine	0.59
Analyzed nutrient composition, %	
Dry matter	88.60
Crude protein	16.23
Ether extract	2.66
Neutral detergent fibre	12.80
Acid detergent fibre	3.77
Calcium	0.54
Total phosphorus	0.55
Gross energy, MJ/kg	16.45

^a^Vitamin-mineral premix supplied the following nutrients per kilogram of diet: vitamin A, 5,512 IU; vitamin D_3_, 2,200 IU; vitamin E, 30 IU; vitamin K_3_, 2.2 mg; vitamin B_12_, 27.6 μg; riboflavin, 4 mg; pantothenic acid, 14 mg; niacin, 30 mg; choline chloride, 400 mg; folic acid, 0.7 mg; thiamine, 1.5 mg; pyridoxine, 3 mg; biotin, 44 μg; Mn (MnO), 40 mg; Fe (FeSO_4_·H_2_O), 75 mg; Zn (ZnO), 75 mg; Cu (CuSO_4_·5H_2_O), 100 mg; I (KI), 0.3 mg; Se (Na_2_SeO_3_), 0.3 mg

^b^SID values were referenced from NRC [77]

The experiment was conducted in 3 consecutive periods (3 pigs per period) using the same facilities and similar experimental procedures because only 3 respiration chambers were available for the study. In each period, pigs were housed individually in metabolism cages and adapted to the cages and diet for 7 d prior to the start of the experimental period. Pigs were fed 2,400 kJ ME/(kg BW^0.6^·d) daily during the adaptation period, which was close to the ad libitum feed intake. The experimental period was 7 d, which was divided into three treatments. The first 5 d were feeding treatment. Pigs were also fed 2,400 kJ ME/(kg BW^0.6^·d) daily. The last 2 d were divided into 24 h fasting and 48 h fasting treatment, respectively. Pigs were fasted with ad libitum access to water via a low-pressure nipple drinker. During the adaptation and experimental period, all pigs were fed the same basal diet and had free access to water. During the experimental period, pigs were housed in metabolism cages that were placed inside open circuit respiratory chambers to determine HP and RQ. The HP of fed state was calculated by averaged the daily HP of the 5-d feeing treatment. The 8-h HP from 22:00 h (d 13) to 06:00 h (d 14) and from 22:00 h (d 14) to 06:00 h (d 15) were calculated and then were extrapolated to a 24-h period, which were considered as the HP of 24 h fasting treatment and 48 h fasting treatments, respectively. Pigs were fed twice daily an equal amount of meal, at 09:00 and 15:30 h. Feed refusals and spillage were recorded daily.

Pigs were weighed at the beginning of adaptation (d 0), and on d 8, 13, 14 and 15, which corresponded to the start or end of the different feeding regimens or fasting times. Temperature in the chambers was maintained at 22 ± 1 °C during the feeding and fasting periods according to Zhang et al. [21].

### Blood collection and animal sampling

The feeding, 24 h fasting and 48 h fasting treatments were terminated around 06:00 h on d 13, 14 and 15, respectively. Three blood samples were collected from the anterior vena cava of each pig at these three time points and represented the blood samples of feeding, 24 h fasting and 48 h fasting treatments. Samples were centrifuged (Heraeus, Hanau, Germany) at 3,000×*g* for 10 min at 4 °C, then the supernatants were transferred to storage tubes, frozen in liquid nitrogen, and stored at − 80 °C for subsequent metabolomics testing.

Urine was collected during the time that pigs were in the respiration chambers according to the methods described by Liu et al. [4]. In brief, urine was collected each morning for each pig in plastic buckets containing 50 mL of 6 N HCl and sieved with cotton gauze, and filtered into a plastic bottle every day. The total quantity of collected urine was weighed and 5% of the daily urinary excretion was stored at − 20 °C. At the end of the experiment, urine collected during the first 5 d was thawed and mixed separately by individual animal and a sub-sample was saved for chemical analysis. Urine was collected separately during the 24 h fasting and 48 h fasting periods. The feed was ground through a 1-mm screen and mixed thoroughly for chemical analysis.

### Chemical analysis and calculation

All chemical analyses of ingredients and diet were conducted in duplicate. Dry matter (DM) was measured by drying the samples in a 135 °C drying oven for 2 h (method 930.15) [22]. The total crude protein (N × 6.25) content of the samples was determined using the Kjeldahl method (method 984.13) [22]. Calcium was measured by titration with 0.1 N KMnO4 (method 927.02) [22]. Total phosphorus was measured colorimetrically using a molybodovanadate reagent (method 965.17) [22]. Ether extract (EE) was determined using the Thiex method [23]. Neutral detergent fibre (NDF) and acid detergent fibre (ADF) were determined using filter bags and fibre analyser equipment (Fibre Analyzer; Ankom Technology, Macedon, NY, USA) following a modification of the procedure of van Soest et al. [24]. The gross energy (GE) in diets was determined using a Parr 6400 bomb calorimeter (Parr Instruments, Moline, IL). The nitrogen in urine were measured according to Liu et al. [4].

Heat production and RQ were calculated daily from CO_2_ and CH_4_ production, O_2_ consumption, and nitrogen excretion in the urine (UN) during the 5 d feeding period and the 2 d fasting period according to the following formulas by Brouwer et al. [25]:

$$ \mathrm{HP}\;\left(\mathrm{kJ}\right)=16.1753\times {\mathrm{O}}_2\left(\mathrm{L}\right)+5.0208\times {\mathrm{CO}}_2\left(\mathrm{L}\right)-2.1673\times {\mathrm{CH}}_4\left(\mathrm{L}\right)-5.9873\times \mathrm{UN}\left(\mathrm{g}\right) $$

$$ \mathrm{RQ}={\mathrm{CO}}_2\left(\mathrm{L}\right)/{\mathrm{O}}_2\left(\mathrm{L}\right) $$

### Sample preparation for metabolomics

Six plasma samples selected randomly from each treatment were extracted using 800 μL ice-cold extraction mix (acetonitrile:methanol, 1:1, *v*:*v*) at a 1:4 sample:extract solution ratio. After vortexing for 5 min, the samples were centrifuged (Eppendorf, Germany) at 15,000×*g* for 10 min at 4 °C for deproteinization. The supernatant fractions were then collected and evaporated to dryness using a vacuum concentrator (Concentrator plus, Eppendorf). The resulting dry residues were re-suspended in 200 μL of methanol:water (4:1), vortexed and centrifuged again at 15,000×*g* for 10 min at 4 °C. Lastly, the supernatant fractions were filtered through a 0.1-μm membrane and transferred to sampler vials to be analyzed on a UPLC-MS system.

### UPLC-MS analysis

Plasma samples were analyzed with an UPLC-HRMS system (UPLC, ACQUITYUPLC H-Class Bio, Waters; MS, Q-Exactive, Termo Scientifc) equipped with a heated electrospray ionization (HESI) source. The UPLC separation was operated on a BEH C18 column (2.1 mm× 100 mm, 1.7 μm, Waters). The HPLC grade solvents and additives from Fisher Scientifc (ThermoFisher Scientific, NJ, USA) were used. Mobile phase was comprised of 0.1% formic acid water solution (A) and 0.1% formic acid ACN solution (B). The gradient program was as follows: 95% A at 0 min to 70% A at 5 min, 5% A at 10 min and held for 3 min, then returned to initial condition. The flow rate was 0.3 mL/min. A sample of pooled plasma was re-injected after each six samples for quality control. The column temperature was set at 35 °C and the injection volume was 5 μL.

The MS analysis was performed in an electrospray ionization positive mode. Key parameters of the HESI source were as follows: spray voltage at 3 kV, capillary temperature at 320 °C, sheath gas flow rate at 30 arb.units, aux gas flow rate at 10 arb. Units, sweep gas flow rate at 5 arb. Units, heater temperature at 350 °C, and s-lens RF level at 55. Full scan data was acquired with a resolution of 70,000 in the mass range of m/z 67.7–1,000. For MS/MS analysis, normalized collision was performed at an energy of 35 V. In addition, an isolation window of 0.8 m/z and a mass resolution of 35,000 were selected.

### Data mining and processing

SIEVE 2.1 software (Thermo Scientific, NJ, USA) was used for metabolomics data processing. This software achieved background subtraction, peak alignment and component extraction of the raw data. Component extraction was performed according to the rules of retention time from 0.5 to 16 min, intensity threshold at 500,000, minimum scan at 9 and signal to noise ratio at 10. Principal components analysis (PCA) was carried out using SIMCA-P 13 software (Umetrics, Umea, Sweden) after data were scaled to Pareto variance. The compounds with *P* <  0.05, fold change > 1.5 and CV < 30% were picked by EXCEL for further identification.

Identification of differential compounds was performed in compound database of METLIN (https://metlin.scripps.edu/landing_page.php?pgcontent=mainPage) and Human Metabolome Database (http://www.hmdb.ca) using accurate mass of molecular ions. The MS/MS spectra database was used to match fragment ion spectra of the candidate compounds. The MS/MS spectra were also compared with theoretical fragmentation patterns with mass accuracy at 5 ppm using Xcalibur™ (Thermo Scientific, NJ, USA).

### Statistical analysis

Data generated in the present experiment were analyzed using the MIXED procedure of SAS (SAS Inst. Inc., Cary, NC) and repeated measurements were considered when analyzed the effects of fasting duration. The statistical model included the main effects of fasting duration. Pig was treated as the experimental unit. Treatment means were separated using the LSMEANS statement and Tukey’s test was used for adjustment in multiple comparison. Results were considered significant at *P* <  0.05 and considered as trends at 0.05 < *P* <  0.10. Boxplot analysis of identified differential compounds were achieved using the R software package (R Development Core Team, 2017,) version 3.4.1). The relative concentrations of differential compounds were imported into Metaboanalyst 3.0 (http://www.metaboanalyst.ca/faces/upload/PathUploadView.xhtml) to generate the metabolome view map, which integrates enrichment analysis and pathway topology analysis. A metabolic pathway which pathway impact value is higher than 0.1 is characterized as the significantly relevant pathways.

## Results

### Indirect calorimetry

Compared to the fed state, the O_2_ consumption decreased at 24 h fasting and continued to decrease at 48 h fasting (*P* <  0.01) (Table 2). The CO_2_ and CH_4_ production of fasted pigs were significant lower than those of fed pigs, while those values were not significantly different between 24 h and 48 h fasting (*P* <  0.01). For HP and RQ, a significant decrease was observed after fasting treatment, but there were not significantly different between 24 h and 48 h fasting (*P* <  0.01). In addition, the UN production tended to decrease with fasting (*P* = 0.054).

**Table 2:** The effect of fasting treatment on heat production, respiratory quotient and urine nitrogen in growing pigs

Items	2400, kJ/(kg BW^0.6^·d)	Fasting		SEM	*P-*value
		24 h	48 h		
*n*	9	9	9		
Body weight, kg	47.6	48.4	46.1	1.9	0.68
Oxygen consumption, L/d	569^a^	417^b^	361^c^	19	< 0.01
Carbon dioxide production, L/d	613^a^	349^b^	294^b^	36	< 0.01
Methane production, L/d	4.5^a^	2.6^b^	1.5^b^	0.4	< 0.01
Heat production, kJ/(kg BW^0.6^·d)	1206^a^	828^b^	733^b^	36	< 0.01
Respiratory quotient	1.07^a^	0.84^b^	0.81^b^	0.01	< 0.01
Urine nitrogen, g/d	9.52	6.83	6.45	0.92	0.054

^abc^Means in the same row with differing superscripts differ (*P* < 0.05)

### Plasma metabolic profiling based on UPLC-HRMS

**Fig. 1:**
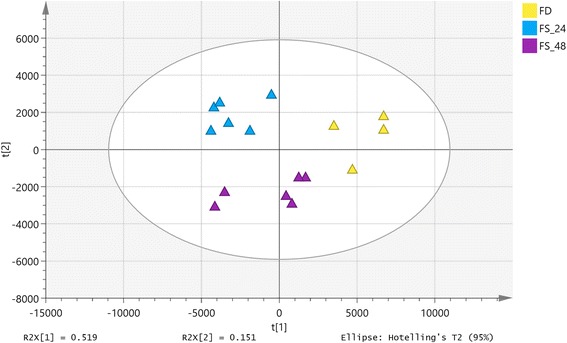
PCA models demonstrating the separation of plasma samples of pigs under feeding, 24 h fasting and 48 h fasting. FD: Feeding; FS_24: 24 h fasting; FS_48: 48 h fasting. Each triangle represents an individual plasma sample

Principal components analysis was performed to reveal intrinsic differences within the signals (Fig. 1). The PCA score plot for plasma of pigs in the fed state, and after 24 h and 48 h of fasting showed clear clustering. PC 1 and PC 2 explained 67% of the total variances within the data. PC1 that described 52% of the variance between the fed and fasted state indicated that there were major differences in the metabolome between these two states, more than between 24 h and 48 h of fasting. The fasted samples differed along PC2 in the PCA score plots. After prolonged fasting, samples moved from the second quadrant to the third quadrant on the score plot, indicating that prolonged fasting alters the composition of the plasma metabolome.

Based on the accurate mass measurement of molecular ions and fragment ions with high resolution, 15 compounds were identified (Table 3). These metabolites belong to different metabolic classes: fatty acid, phospholipid, amino acids, and choline. Fold change was calculated by dividing the mean of normalized intensity of each plasma metabolite in the former by the mean of normalized intensity of each plasma in the latter. A fold change > 1 indicates that the metabolite was down-regulated, whereas a fold change < 1 indicates the metabolite was up-regulated. To illustrate the direction of changes at different time points, data were visualized in the form of box-plots.

**Table 3:** Metabolites with significant differences among feeding, 24 h of fasting and 48 h of fasting^a^

No.	Name	m/z	Formula	Fold change^b^			Pathway analysis
				FD/FS_24	FD/FS_48	FS_24/FS_48	
1	12,13-DHOME	297.2420	C_18_H_34_O_4_	0.30	0.49	1.62	Linoleic acid metabolism
2	Linoleic acid	263.2366	C_18_H_32_O_2_	0.17	0.20	1.18	Linoleic acid metabolism
3	Stearidonic acid	277.2157	C_18_H_28_O_2_	0.11	0.11	1.02	alpha-Linolenic acid metabolism
4	Oleic acid	300.2893	C_18_H_34_O_2_	0.38	0.62	1.62	Fatty acid metabolism
5	Palmitoleic acid	255.2314	C_16_H_30_O_2_	0.21	0.25	1.17	Fatty acid metabolism
6	Pantothenic acid	220.1178	C_9_H_17_NO_5_	1.22	0.72	0.59	Pantothenate and CoA biosynthesis
7	Glycerophosphocholine	280.0917	C_8_H_20_NO_6_P	1.75	1.51	0.86	Ether lipid metabolism
8	LysoPC(O-18:0)	510.3912	C_26_H_56_NO_6_P	1.02	0.34	0.33	Ether lipid metabolism
9	Sphinganine	284.2942	C_18_H_39_NO_2_	0.37	0.46	1.24	Sphingolipid metabolism
10	5-Aminopentanoic acid	118.0864	C_5_H_11_NO_2_	1.30	0.84	0.65	Lysine degradation
11	Aminoadipic acid	144.0654	C_6_H_11_NO_4_	1.32	0.83	0.63	Lysine degradation
12	Betaine	140.0681	C_5_H_11_NO_2_	1.87	1.05	0.56	Glycine, serine and threonine metabolism
13	Ornithine	133.0972	C_5_H_12_N_2_O_2_	2.00	1.64	0.82	Arginine and proline metabolism
14	*L*-Glutamine	169.0582	C_5_H_10_N_2_O_3_	2.12	1.66	0.78	Arginine and proline metabolism
15	*L*-Tyrosine	199.1076	C_9_H_11_NO_3_	0.49	0.45	0.93	Nitrogen metabolism

^a^FD: Feeding; FS_24: 24 h fasting; FS_48: 48 h fasting; 12,13-DHOME: 12,13-dihydroxyoctadecenoic acid; LysoPC(18:0): lysophosphatidylcholine 18:0

^b^Fold change was calculated by dividing the mean of normalized intensity of each plasma metabolite in the former by the mean of normalized intensity of each plasma in the latter. Fold change > 1 indicates that the metabolite was down-regulated, whereas fold change < 1 indicates the metabolite was up-regulated

**Fig. 2:**
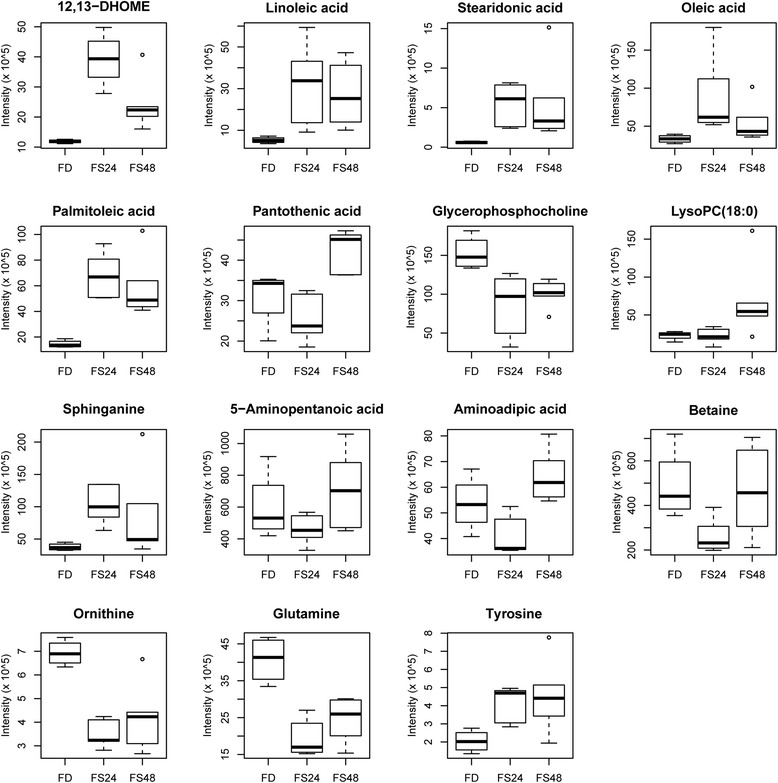
Identified compounds that change during feeding, 24 h fasting and 48 h fasting. Relative concentrations of identified compounds are presented on the *Y*-axis. Time points of sampling are presented on the *X*-axis and are defined as follows: FD: Feeding; FS24: 24 h fasting; FS48: 48 h fasting. 12,13-DHOME: 12,13-dihydroxyoctadecenoic acid; LysoPC(18:0): lysophosphatidylcholine 18:0

Compared to the fed state, plasma 12,13-dihydroxyoctadecenoic acid (12,13-DHOME) and sphinganine levels significantly increased after fasting, slightly at 48 h of fasting and moderately at of 24 h fasting (Fig. 2). In accordance with the feeding stage, linoleic acid, stearidonic acid, oleic acid, palmitoleic acid, and tyrosine levels were low before fasting but increased and showed much larger variability after 24 h or 48 h of fasting. Also, pantothenic acid, lysophosphatidylcholine 18:0 (LysoPC (18:0)) and 5-aminopentanoic acid levels were higher at 48 h of fasting compared to those at 24 h of fasting. Not all metabolites were up-regulated during fasting. Levels of glycerophosphocholine (GPC), ornithine, and glutamine were significantly higher in the fed state compared to those at 24 h or 48 h of fasting. Plasma aminoadipic acid and betaine levels were similar in the fed state and at 48 h of fasting, but were lower at 24 h of fasting showed.

**Fig. 3:**
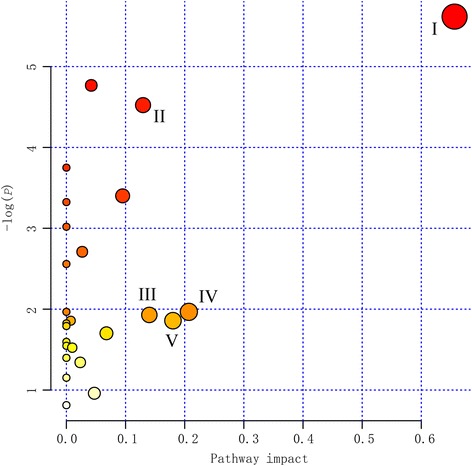
Topology analysis of metabolic pathways identified among the feeding, 24 h fasting and 48 h fasting comparisons. The *X*-axis represents the pathway impact, and *Y*-axis represents the pathway enrichment. Larger sizes and darker colors represent greater pathway enrichment and higher pathway impact values, respectively. I: Linoleic acid metabolism; II: Arginine and proline metabolism; III: Sphingolipid metabolism; IV: Alanine, aspartate and glutamate metabolism; V: Pantothenate and CoA biosynthesis

Pathway enrichment and pathway topology analysis were performed using MetaboAnalyst 3.0, which based on high-quality KEGG metabolic pathways as the backend knowledgebase (Fig. 3). Based on the identified metabolites and changes in their concentrations, five metabolic pathway had a pathway impact value higher than 0.1, which is the cutoff value for relevance. The five significantly relevant metabolic pathways that indicated the growing pigs’ responses to fasting treatment included: linoleic acid metabolism, pantothenate and CoA biosynthesis, arginine and proline metabolism, alanine, aspartate, and glutamate metabolism, and sphingolipid metabolism.

## Discussion

The term “indirect calorimetry” refers to the fact that heat production is determined by measuring O_2_ consumption, and CO_2_ and CH_4_ production, which are indicative for the oxidation of metabolites [26]. During fasting, these substrates are mainly glucose, lipids, and proteins mobilized from body reserves [27]. In the present study, heat production during fasting was significantly lower than that in the fed state, which indicates that the oxidation of substrate is down-regulated. Heat production at 24 and 48 h of fasting was 823 and 733 kJ/(kg BW^0.6^·d), respectively. These values are within the range of fasting HP from 700 to 955 kJ/(kg BW^0.6^·d), which were determined in previous studies [28–31]. The variation might be partly explained by differences in the duration of fasting. The RQ is indicative for macronutrients that are being oxidized, as oxidation of glucose, lipids, and proteins yield different RQ values [32]. In the present study, the RQ at 24 and 48 h of fasting were 0.84 and 0.81, respectively. A value of 0.7 indicates that lipids are being catabolized, 0.8 for proteins, and 1.0 for glucose [33]. The only occurrence of glucose oxidation in the body can be reflected in RQ values equal to 1.0 [34]. An RQ below 1.0 indicates that lipids and protein are being catabolized [35]. Therefore, the RQ of 0.84 and 0.81 indicates that mainly lipids and protein were oxidized during fasting [36, 37]. In addition, indirect calorimetry analysis revealed that the HP, RQ and UN of growing pigs were no significant difference between 24 h fasting and 48 h fasting. These results can be explained that the energy metabolism of growing pigs under a 2-d fasting treatment was relatively stable, which were in agreement with the reports of Liu et al. [4] who also measured HP of fasted pigs using indirect calorimetry. Based on these results, a 24-h fasting treatment was more appropriate than a 48-h fasting treatment to determine the effect of fasting on the energy metabolism of growing pigs.

The body’s principal lipid classes are mainly triglycerides, phospholipids, and steroids but triglycerides are quantitatively the most important lipids [38, 39]. Humans and animals will break down lipids to meet their energy requirements when the energy supply is restricted. This is a strategy to save glucose and protein that are crucial fuels for some important organs [40]. A series of studies have reported the effect of feed restriction and fasting on lipid metabolism [41–43]. Compared to biochemical traits in the blood of lipid metabolism, metabolomics provides new and a more in-depth information of the global metabolite profile of plasma [44, 45].

As mentioned above, five metabolites of unsaturated fatty acids (i.e., 12,13-DHOME, linoleic acid, stearidonic acid, oleic acid and palmitoleic acid were significantly up-regulated during fasting. Similar results have been reported in other studies in animals [13]. In the fed state, unsaturated fatty acids, saturated fatty acids, and glycerol are used to synthesize triglycerides [46]. During fasting, fatty acids are released from triacylglycerol stored in adipocytes of growing pigs, resulting in increased levels in plasma [47]. Wood et al. [48] concluded that more than half of the fatty acids in animal lipids were unsaturated fatty acids, and unsaturated fatty acids were oxidized more rapidly than saturated fatty acids [49, 50]. The least oxidized of the saturated fatty acids having the greatest retention in the carcass [51]. In addition, multiple transcripts involved in the pathway of saturated fatty acids synthesis were inhibited in rat adipose tissue after short-term fasting [11]. Thus, the unsaturated fatty acids were more efficient energy substrate than saturated fatty acids, which may be explained that unsaturated fatty acids were primarily identified in the current study. Compared with the fed state, the fold change in the level of unsaturated fatty acids at 24 h and 48 h of fasting was close to 1, which indicates that the level of unsaturated fatty acids level was not significantly different at 24 h and 48 h of fasting (Table 3). The pathway of linoleic acid metabolism was identified as the most significant pathway through pathway topology analysis (Fig. 3). On the whole, the change of unsaturated fatty acid primarily contribute to fasting metabolism in our study and suggested that 24 h of fasting was more appropriate than 48 h of fasting for indicators of metabolic components of fasting metabolism.

In contrast, the GPC concentration was found to be significantly down-regulated during fasting. The GPC is a choline derivative that functions as a substrate in many bio-metabolic pathways [52]. As we know, the GPC is formed during the breakdown of phosphatidylcholine and is part of a phospholipid pathway that is active in many body tissues [53]. In the present study, fasting induced a significant decrease in the relative concentration of GPC, while the GPC level was not affected by the duration of fasting. Some studies have shown that the GPC level can be indicative for the ability to break down phospholipids as a fatty acid source to meet the energy requirements [54, 55]. Low GPC values can be accompanied by a high level of ketone bodies, which are produced by the liver from fatty acids and serve as an energy source for tissues during starvation or prolonged exercise [56, 57]. A recent study by Klein et al. [55] suggested that GPC could be used as a prognostic method for the risk of ketosis. Therefore, a decline of GPC during fasting reflects a switch of energy metabolism from phospholipids to fatty acid oxidation when the available energy is limited [55]. In addition, the almost constant levels of GPC during 24 and 48 h of fasting indicates that phospholipids metabolism of growing pigs was relatively stable during fasting.

In addition to triglycerides, phospholipids and sphingolipids are also involved in lipid metabolism. Among the identified compounds of the current study, pantothenic acid and LysoPC (18:0) participated in phospholipid metabolism. Most of these metabolites showed a significant increase during the 48 h of fasting [58, 59]. Pantothenic acid is an essential vitamin and required precursor for the biosynthesis of coenzyme A (CoA) in mammalian tissue [59]. Reibel et al. [60] reported that fasting resulted in higher tissue concentrations of Pantothenic acid, increased incorporation of Pantothenic acid into CoA, and elevated tissue concentrations of CoA in the liver. The CoA may act as an acyl group carrier in all living organisms, where it diverse cellular functions as an indispensable cofactor in energy metabolism, including the oxidation of fatty acids, carbohydrates, pyruvate, ketone bodies, and amino acids [61, 62]. The increase of Pantothenic acid can be explained that the interconversion of Pantothenic acid and CoA in the tissue was accelerated in vivo by prolonged fasting to meet the energy requirement [63].

Interestingly, we found a significant decrease in the level of betaine at 24 h of fasting. Betaine has a main physiological function as a methyl donor in the (re)formation of methionine from homocysteine [64]. A recent study of Rubio-Aliaga et al. [6] showed that methionine levels declined over 50% during fasting. The significant decline of betaine at 24 h fasting may be explained that betaine actively participates in the methionine cycle, which is a methyl donor taking part in several highly important methylation reactions [65]. However, the unexpected increase of betaine at 48 h of fasting may be explained by the observation that betaine can be maintained endogenously from choline, a process that occur mainly in the mitochondria of liver and kidney cells [66, 67].

Ornithine is a non-proteinogenic amino acid that plays an indispensable role in the urea cycle [68, 69]. In the current study, fasting induced a significant decrease in the relative concentration of ornithine, whereas ornithine levels were not affected by prolonged fasting. The change in urinary nitrogen concentration coincided with the changes in ornithine level, which indicated that ornithine was intimately associated with urinary nitrogen excretion as an important intermediate of the urea cycle in mammals [70]. Similarly, glutamine concentrations were also decreased during fasting. Rubio-Aliaga et al. [6] reported that glutamine showed negative correlations with prototypical markers of fasting such as NEFA. Glutamine is an abundant amino acid in the plasma where it functions as a non-toxic nitrogen vehicle and a respiratory fuel [71]. The decline of glutamine may partly be explained by its role as a glucose precursor during fasting by providing carbon for gluconeogenesis [16, 72]. The pathway topology analysis identified that ornithine and glutamine were enriched in the arginine and proline metabolic pathway. Recent studies reported by Pang et al. [69] indicated that proline catabolism was associated with lipid utilization by transcription factor SKN-1 during fasting. We speculated that there was a potential interaction between amino acid and lipid metabolism through those identified metabolites.

Tyrosine is not only a conditionally essential amino acid, but also a potent ketogenic amino acid [73]. Ketone bodies are formed in the liver and contributed as a fuel during fasting [74]. The level of tyrosine was significantly up-regulated during fasting in the current study. The increase of tyrosine may partly be explained by tyrosine acting as a ketogenic amino acid to meet the energy requirements of growing pigs during fasting [75, 76]. In addition, compared with amino acids level in the fed state, the fold change of tyrosine, ornithine and glutamine level at 24 h and 48 h of fasting were close to 1, which indicated that amino acids level were not significantly different at 24 h and 48 h of fasting.

## Conclusions

In conclusion, a differential compound library containing 15 metabolites was identified that contributed to the differences in HP and RQ between the fed and the fasted state in growing pigs. Integrative analysis of indirect calorimetry and metabolomics profiling revealed that the decreases in HP and RQ of growing pigs under fasting conditions were associated with the alterations of linoleic acid metabolism and amino acid metabolism. The integrative analysis also indicated that growing pigs under a 24-h fasting were more appropriate than a 48-h fasting to investigate the metabolic components of maintenance energy expenditure. Our findings help to improve knowledge regarding potential mechanisms responsible for metabolic components of maintenance energy expenditure and provide possible important implications for the design of effective strategies to study fasting mechanisms.

## Abbreviations

ADF: Acid detergent fibre
DHOME: Dihydroxyoctadecenoic acid
DM: Dry matter
EE: Ether extract
GE: Gross energy
GPC: Glycerophosphocholine
HESI: Heated electrospray ionization
HP: Heat production
LysoPC: Lysophosphatidylcholine
NDF: Neutral detergent fibre
NEFA: Nonesterified fatty acid
PCA: Principal component analysis
RQ: Respiratory quotient
UN: Nitrogen excretion in urine
